# Exploring the therapeutic potential of recombinant human lysozyme: a review on wound management system with antibacterial

**DOI:** 10.3389/fbioe.2023.1292149

**Published:** 2023-11-01

**Authors:** Meiping Zhao, Meili Huang, Zhen Li

**Affiliations:** ^1^ Nursing Department, Sir Run Run Shaw Hospital, Zhejiang University School of Medicine, Hangzhou, China; ^2^ Nursing Department, Sir Run Run Shaw Hospital Affiliated to Zhejiang University School of Medicine Alar Hospital, Alar, China; ^3^ Emergency Department, Sir Run Run Shaw Hospital, Zhejiang University School of Medicine, Hangzhou, China

**Keywords:** lysozyme, wound healing, antibacterial, wound dressing, hydrogels

## Abstract

Lysozyme, a natural antibacterial enzyme protein, possesses the ability to dissolve the cell walls of Gram-positive bacteria, demonstrating broad-spectrum antibacterial activity. Despite its significant potential in treating wound infections and promoting wound healing, its widespread clinical application has yet to be realized. Current research is primarily focused on carrier-based delivery systems for lysozyme. In this review, we discuss four delivery systems that can be employed for lysozyme in wound healing treatment, specifically hydrogels, nanofilms, electrospun fibrous membranes, and modified-lysozyme composite systems. These systems not only enhance the stability of lysozyme but also enable its controlled and sustained release at wound sites, potentially overcoming some of the challenges associated with its direct application. Lastly, we delve into the perspectives and challenges related to the use of these delivery systems, hoping to spur further research and innovation in this promising field.

## 1 Introduction

Wound healing is a complex and essential physiological process that is fundamental to human health (Edwards and Keith, 2004). It involves a series of coordinated events, including inflammation, proliferation, and remodeling (Monaco and Thomas Lawrence, 2003; Harper et al., 2014; Qing, 2017). This process is an intricate balance of cellular and biochemical events that work in concert to restore the structural and functional integrity of the skin (Han and Roger, 2017; Wang et al., 2022). However, this delicate balance can be easily disturbed by a variety of factors, such as infections and chronic conditions like diabetes, leading to non-healing or chronic wounds (Harding et al., 2002; Demidova-Rice et al., 2012; Morton and Tania, 2016). Poor nutrition can also play a role in hampering the wound healing process (Stechmiller, 2010).

The global burden of chronic wounds is considerable, with significant implications for individuals and healthcare systems (DesJardins-Park et al., 2022; Sadeghi-Aghbash et al., 2023; Zhang et al., 2023). Chronic wounds can drastically impact the quality of life of affected patients, leading to physical discomfort and emotional distress (Frykberg Robert and Banks, 2015; Morton and Tania, 2016). Furthermore, they place a substantial strain on healthcare resources, necessitating prolonged treatment and care (Nunan et al., 2014; Powers et al., 2016).

Recombinant human lysozyme (rhLZM), an enzyme first discovered by Alexander Fleming in 1922, has been widely recognized for its pivotal role in the innate immune system (Roh et al., 2006; Nunan et al., 2014). Found in a diverse range of organisms from bacteria to humans, rhLZM is particularly abundant in human body secretions such as tears and saliva, standing as a first line of defense against microbial invasion in these areas of the body (Klockars and Reitamo, 1975; Vårum et al., 1997). The primary function of rhLZM lies in its potent ability to break down the polysaccharide components of bacterial cell walls (Ellison and Giehl, 1991; Gao and Fallon, 2000; Ragland and Alison, 2017). This enzymatic action leads to cell lysis, causing the invading bacterial cells to rupture and die, thus playing an essential role in the body’s defense against bacterial infections (Wu et al., 2018). This inherent bacteriolytic, or bacteria-destroying, property of rhLZM makes it an attractive candidate for applications in managing bacterial infections commonly associated with wounds (Stechmiller, 2010; Gong et al., 2022). However, the role of rhLZM in human physiology extends beyond merely protecting against bacterial invasions (Frykberg Robert and Banks, 2015). It is also involved in inflammation and has a role in tissue remodeling, both of which are crucial elements of the wound healing process (Jones et al., 2003; Bhattacharyya et al., 2010). These multifunctional characteristics have motivated research into the potential application of rhLZM in wound management.

Despite its promising therapeutic potential, the application of lysozyme alone is restricted in several ways. 1) When applied directly to the wound surface, lysozyme can be easily washed away by exudate, resulting in a short retention time (Klockars and Reitamo, 1975; Roh et al., 2006). 2) Lysozyme is prone to degradation by proteases present in body fluids, leading to poor stability (Vårum et al., 1997). 3) Lysozyme also has some immunogenicity, and repeated use may induce immune responses (Gao and Fallon, 2000; Ragland and Alison, 2017). 4) Additionally, lysozyme exhibits weaker antimicrobial effects against Gram-negative bacteria compared to Gram-positive bacteria (Ellison and Giehl, 1991). This differential antimicrobial activity can limit its overall effectiveness in combating a broad spectrum of bacterial infections.

In the face of these challenges, innovative therapeutic delivery approaches have been developed to enhance the therapeutic efficacy of lysozyme while minimizing side effects (Wu et al., 2018). These include hydrogels, nanofilms, electrospun fibrous membranes, and modified-lysozyme composite systems (Table 1) (Gong et al., 2022). These materials can improve the stability of lysozyme, reduce the immunogenicity of lysozyme, and thereby reduce immune responses *in vivo* (Jones et al., 2003; Bhattacharyya et al., 2010). Sustained release of lysozyme from delivery systems increases its retention time at the wound site, prolongs its antibacterial action, and prevents rapid degradation (Bhattacharyya et al., 2010).

**TABLE 1:** Therapeutic delivery systems for lysozyme in wound healing.

Delivery system	Advantages	Challenges	References
Hydrogels	1. Controlled drug release 2. Protection of lysozyme 3. Moisture maintenance 4. Absorption of exudates 5. Comfort and conformability	1. Control and optimization of lysozyme loading and release dynamics 2. Harmonizing hydrogel degradation rate with wound healing progression	Ma et al. (2011), Xu et al. (2018), Tan et al. (2019), Chen et al. (2022b), Wang et al. (2023b), Chen et al. (2023)
Nanofilms	1. High lysozyme loading. 2. Precise dosage control. 3. Direct lysozyme delivery and patient comfort. 4. Bacterial barrier and continuous wound monitoring	1. Fabrication of nanofilms requires optimization of materials and techniques. 2. Preservation of lysozyme’s bioactivity. 3. Sustained release of bioactive lysozyme in synergy with other antimicrobials. 4. Biocompatibility and biosafety	Ohno and David (1989), Tenovuo (2002), Charernsriwilaiwat et al. (2012), Liu et al. (2013), Wang et al. (2017), Liu et al. (2018), Wu et al. (2020), Tian et al. (2022), Zhou et al. (2023)
Electrospun Fibrous Membranes	1. High surface area for high lysozyme loading capacity. 2. Interconnected pore structure for efficient nutrient transport, waste removal, and cell infiltration. 3. Nanotopography and extracellular matrix beneficial for cell adhesion and growth. 4. Direct use as wound dressings due to flexibility, gas permeability, and biocompatibility	1. Scale-up manufacturing of uniform fibrous matrices. 2. Encapsulation and stabilization of lysozyme activity. 3. Sustained release of bioactive lysozyme over clinically relevant timescales	Branchu et al. (1999), Chiu et al. (2011), de Oliveira et al. (2012), Briggs et al. (2015), Agarwal et al. (2017), Li et al. (2017), De France et al. (2020), Kim et al. (2020), Wu et al. (2021), Zhao et al. (2022)
Modified-lysozyme composite	1. Biomacromolecule provides a protective shell to lysozyme, shielding it from degradation. 2. Controlled release of lysozyme over time. 3. Biocompatibility and biodegradability of many biomacromolecules. 4. Enhanced effectiveness of lysozyme through functionalization to target specific cells or tissues. 5. Potential reduction in required dosage and associated side effects	1. Regulation and sustained release of lysozyme. 2. Maintaining stability and activity of lysozyme within the complex. 3. Biocompatibility and safety of the complex need thorough evaluation. 4. Process development for manufacturing these complexes is complex and needs to be reproducible, scalable, and cost-effective	De Vita et al. (1984), Swaminathan et al. (2011), Woods et al. (2011), Gudipaneni et al. (2014), Zou et al. (2019)

Recent advances in biotechnology have led to the development of these innovative lysozyme delivery systems (Chaudhary et al., 2020; Wang et al., 2023a). This review aims to provide an overview of the current knowledge surrounding the application of these various delivery systems, including hydrogels, nanofilms, electrospun fibrous membranes, and modified-lysozyme composite systems. We will delve into their respective advantages and limitations, discuss their mechanisms of action, and examine the existing body of research on this topic. By doing so, we hope to inspire further research and innovation in this promising field, ultimately leading to improved wound care strategies and better patient outcomes.

## 2 Fermentation production and therapeutic applications of recombinant human lysozyme

Recombinant human lysozyme (rhLZM) is a promising antimicrobial and immunomodulatory protein that has attracted significant interest for its therapeutic potential. In recent decades, there have been notable advancements in recombinant DNA technology and fermentation techniques, leading to improved production yields and purity of rhLZM (Yu et al., 2014; Ercan and Ali, 2016).

*Escherichia coli* remains the predominant host for heterologous expression of rhLZM. Strategies such as codon optimization, co-expression of chaperones, and protease knockouts in *E. coli* have significantly enhanced volumetric yields, reaching up to 1.5 g/L (Zavaleta et al., 2007). By employing high-cell density fed-batch fermentations under optimized conditions, *E. coli* can efficiently accumulate rhLZM. *Bacillus subtilis* is another attractive host due to its Generally Recognized As Safe (GRAS) status, secretion capabilities, and lack of endotoxins. Engineered *B. subtilis* strains with increased protease deficiency and simplified downstream processing have achieved rhLZM titers of approximately 500 mg/L (Dai et al., 2015). Yeast systems, such as *Pichia pastoris* and *Saccharomyces cerevisiae*, are also gaining traction for secreting rhLZM with yields exceeding 1 g/L (Symmank et al., 2002). Compared to prokaryotes, yeasts can provide proper protein folding and post-translational modifications.

Innovative fermentation strategies, including the addition of chemical chaperones, temperature or pH shifts, controlled oxygen limitation, and fed-batch glucose feeding, have improved the productivity of rhLZM across various expression platforms (Tong et al., 2011; Liu et al., 2012). Two-stage processes that separate growth and production phases allow precise control over culture parameters, leading to enhanced rhLZM folding and reduced protease degradation. High-cell density cultures induced in the stationary phase often yield several-fold higher rhLZM than conventional batch processes (Yu et al., 2014). Continuous fermentation for extended durations, with dynamically controlled cell densities, inducer levels, and media feed rates, has emerged as an efficient manufacturing strategy.

**FIGURE 1:**
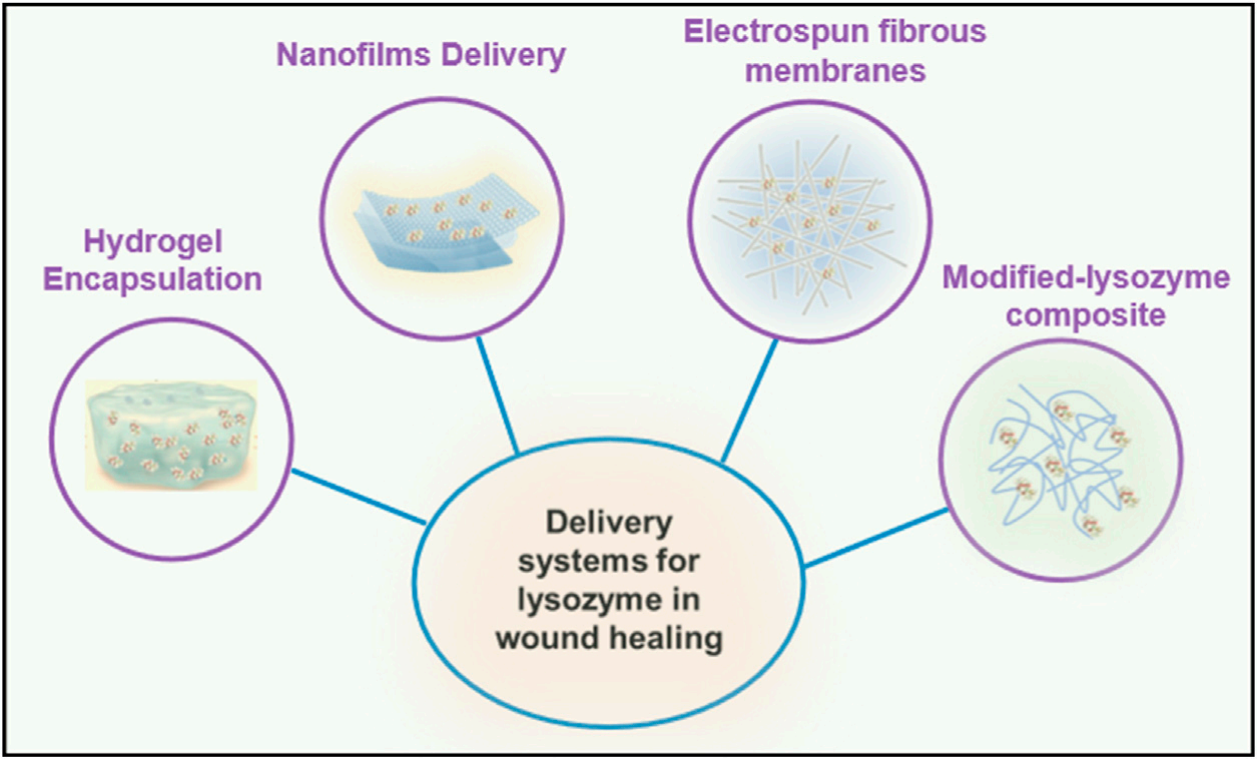
Four kinds of delivery systems for lysozyme in wound healing.

Novel expression systems, such as the methyloptrophic bacteria *Pseudomonas* methanica and methylotrophic yeasts, are being explored as alternatives to traditional hosts. These organisms can achieve very high biomass on methanol, potentially resulting in elevated yields (Dam et al., 2013). Membrane-based processes that integrate reaction and separation, such as extractive fermentations or *in situ* product removals, have shown promise in lowering product inhibition and proteolysis, thereby improving rhLZM stability. The proteolytic activities of rhLZM aid in the detachment of dead tissue and degradation of biofilm matrices, promoting faster debridement and disinfection of chronic wounds (Vårum et al., 1997). Furthermore, rhLZM stimulates re-epithelialization, angiogenesis, and collagen deposition, thereby facilitating faster wound closure. Compared to conventional antibiotics, rhLZM is less likely to induce resistance due to its multifaceted mechanisms of action (Ferraboschi et al., 2021). However, the high production cost of pure rhLZM remains a significant challenge for practical wound dressings. Researchers are investigating the encapsulation of rhLZM in nanoparticle carriers and its integration with scaffold materials to enhance delivery efficiency and enable sustained topical delivery throughout the healing period (Alves et al., 2017). Nevertheless, potential concerns, such as burst release, low loading efficiency, and diminished bioactivity, need to be addressed with current carrier systems for rhLZM. Further research on novel delivery vehicles and scaffold formulations is necessary to fully unlock the healing potential of rhLZM while overcoming its delivery limitations (Figure 1).

## 3 Hydrogels

Hydrogels are highly absorbent, three-dimensional networks of hydrophilic polymers that can hold a substantial amount of water or biological fluids (Roh et al., 2006; Chen et al., 2022a; Wang et al., 2023a). Hydrogels can absorb wound exudates, reducing inflammation and the risk of infection (Gong et al., 2023). Their soft and flexible nature allows them to conform to the wound shape, providing protection and comfort. They are formed by the crosslinking of hydrophilic polymers, which can be either synthetic or natural in origin (Yang et al., 2018; Gong et al., 2023). The crosslinking can be achieved through various physical or chemical methods. Physical crosslinking involves methods such as ionic crosslinking, crystallite formation, or hydrogen bonding, while chemical crosslinking generally involves covalent bonds formed during polymerization reactions (Zhao et al., 2020; Gong et al., 2022). The degree of crosslinking in the hydrogel network affects its properties like mechanical strength, elasticity, and swelling capacity. Meanwhile, they have high water content, which provides a moist environment that is conducive for wound healing.

The application of hydrogels in delivering lysozyme for wound healing leverages on their unique properties: 1) Controlled drug release: hydrogels can encapsulate lysozyme within their network and release it in a controlled manner over time. This allows for a sustained presence of lysozyme at the wound site, enhancing its therapeutic efficacy (Wang et al., 2023b; Chen et al., 2023; Gong et al., 2023); 2) Protection of lysozyme: the hydrogel matrix can protect lysozyme from degradation by proteases present in wound exudates, thereby preserving its activity (Tan et al., 2019); 3) Moisture maintenance: by maintaining a moist wound environment, hydrogels promote the wound healing process. This environment is conducive to cell migration, proliferation, and enzymatic activity (Pangburn et al., 1982); 4) Absorption of exudates: hydrogels can absorb wound exudates, reducing the risk of wound maceration and infection (Chen et al., 2022a). 5) Comfort and Conformability: due to their soft and flexible nature, hydrogels can conform to the wound shape, providing effective coverage and patient comfort (Yang et al., 2019).

### 3.1 Gallic acid hydrogel

**FIGURE 2:**
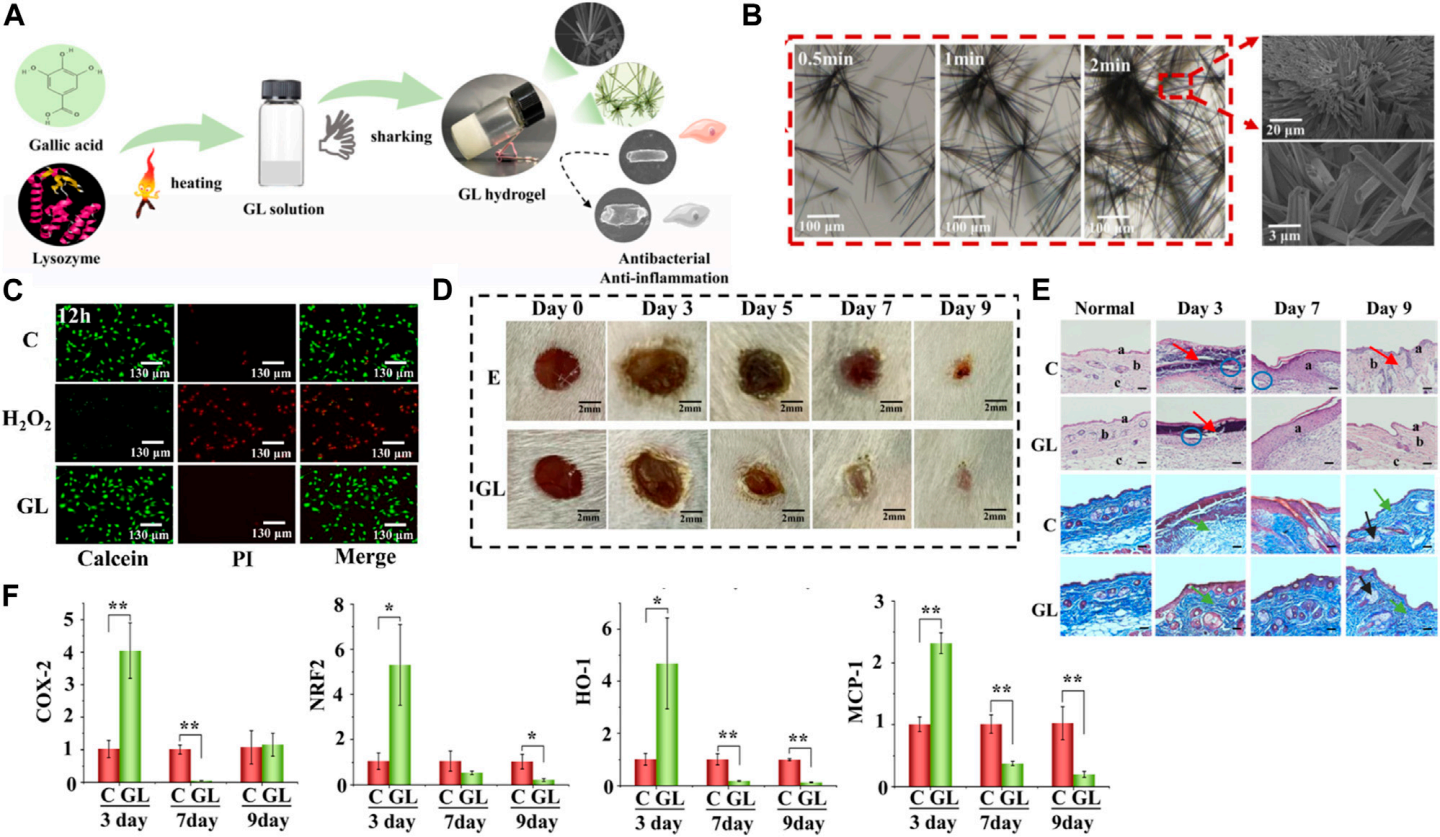
**(A)** Schematic representation illustrating the self-assembly process involved in the formation of the GL hydrogel. **(B)** Microscopic images capturing the evolution of microfiber structure at intervals of 0.5, 1, and 2 min. Scale bar, 100, 20, and 3 μm. **(C)** Visualization of live/dead 3T3 cells following a 12-h incubation with the GL hydrogel, using Calcein-tracker green staining for live cells and propidium iodide for dead cells; hydrogen peroxide treatment serves as a positive control. **(D)** Representative-images along with the corresponding wound areas for E (bacteria-infected wounds) and GL (bacteria-infected wounds post-GL hydrogel treatment) on days 0, 3, 5, 7, and 9. Scale bar, 2 mm. **(E)** Histological micrographs of skin tissues from **(C)** (wound treated with normal saline) and GL (wound post-GL hydrogel treatment) groups on days 3, 7, and 9, stained with Hematoxylin and Eosin (H&E) and Masson. The scale bar indicates a length of 100 μm. **(F)** mRNA expression levels in skin tissues obtained from the control group or the GL hydrogel-treated group on days 3, 7, and 9. Reproduced with permission from Wu et al. (2018).

In a study, gallic acid and lysozyme were used to construct slow-release hydrogels for wound treatment (Gong et al., 2022). The hydrogel was designed to enable the spatiotemporal delivery of gallic acid and lysozyme, two natural products with excellent wound-healing properties (Figure 2A). The research aimed to investigate the properties of the hydrogel, including its biocompatibility, antibacterial activity and wound healing effects.

The hydrogel utilizes gallic acid and lysozyme mixed with the thermosensitive polymer Pluronic F127. Scanning electron microscopy (SEM) results showed that the hydrogel had a porous structure, good thermal stability, and excellent swelling properties (Figure 2B). *In vivo* experimentation utilizing a rat model provided evidence that the hydrogel displays commendable biocompatibility and does not elicit any deleterious effects on the skin. Evaluation of cellular viability via live/dead staining demonstrated that, following a 12-h treatment, GL hydrogels did not inflict substantial harm on 3T3 fibroblasts, thereby suggesting the absence of discernible damage induced by the hydrogel (Figure 2C). The antibacterial activity of the hydrogels was evaluated using the disk diffusion method and minimum inhibitory concentration (MIC) assay. The results showed that the hydrogel had excellent antibacterial activity against *E. coli* (Figure 2D). Evaluating the wound healing effects of hydrogels using a rat model. The results showed that the hydrogel significantly accelerated the wound healing process, with faster wound closure, greater collagen deposition, and a lower inflammatory response (Figures 2E, F). The authors claim that this straightforwardly prepared GL hydrogel can be utilized as an outstanding wound dressing in clinical settings. Their study not only presents a method for co-assembling these naturally bioactive compounds but also expands their applications in biomedical materials.

This study suggests a new strategy to create temperature-controlled hydrogels with excellent wound-healing properties. The hydrogel was prepared from natural products, gallic acid, and lysozyme, and exhibited good biocompatibility, antibacterial activity, and wound healing. The results of this study show that hydrogels have great potential for treating various types of wounds.

### 3.2 Gelatin hydrogel

In 2022, Wang et al. (2023a) have pioneered a novel, dual-layer hydrogel dressing. Their goal is to amplify the therapeutic impact and expedite the repair process for severe, biofilm-infected chronic wounds. The dressing is a concoction of GelMA/gelatin and incorporates Epidermal Growth Factor (EGF) as well as lysozyme-loaded mesoporous polydopamine (MPDA) nanoparticles. These MPDA nanoparticles have excellent biocompatibility and possess a robust capacity to transform light into heat. Their mesoporous design provides a larger surface area, making for a more effective drug-loading and delivery system. Research results indicate that these nanoparticles can accommodate an increased quantity of antibacterial agents, thereby boosting the photothermal effect and compensating for the inadequacies of low-temperature photothermal therapy (PTT). For the effective elimination of biofilms, the MPDA nanoparticles are loaded with lysozyme. These nanoparticles’ photothermal effect disrupts the biofilms’ structure, allowing the loaded lysozyme to deeply penetrate the biofilms and exterminate the bacteria.

**FIGURE 3:**
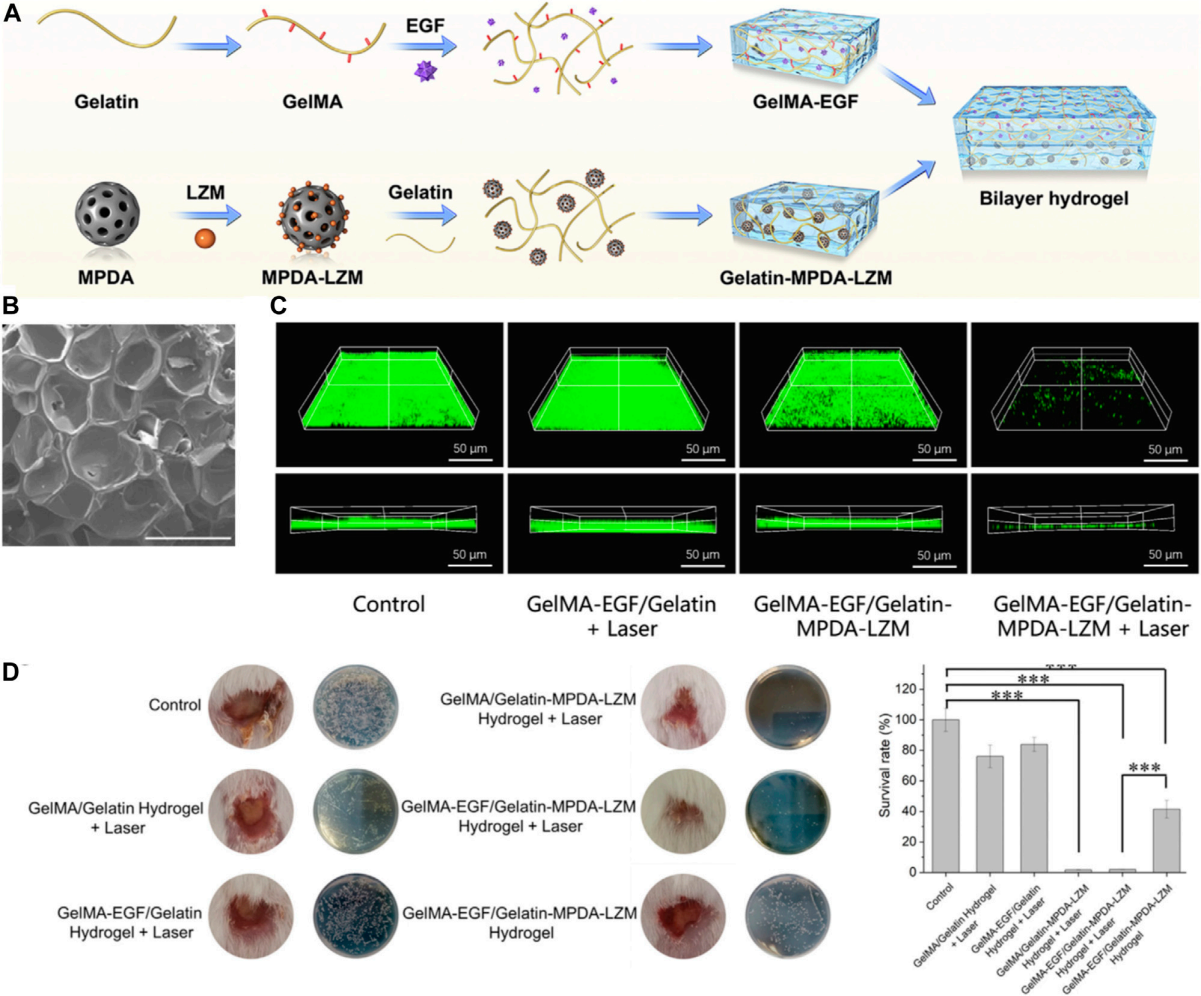
**(A)** Schematic depiction of the preparation procedure for the GelMA-EGF/Gelatin-MPDA-LZM bilayer hydrogel dressing. **(B)** Scanning Electron Microscopy (SEM) images of GelMA. The scale bar represents 500 μm. **(C)** Three-dimensional (3D) Confocal Laser Scanning Microscopy (CLSM) images of SYTO 9 stained *E. coli* biofilms following different treatments. The scale bar represents 50 µm. **(D)** Quantitative analysis of **(B)** Interleukin-1 beta (IL-1β) levels, **(C)** Representative photographs of wounds treated with various hydrogel systems for 4 days, accompanied by a quantitative measurement of bacterial survival rate based on Colony Forming Unit (CFU) counts in standard plate assays (*n* = 3, ***p < 0.001). Reproduced with permission from Chaudhary et al. (2020).

The hydrogel dressing is innovatively designed as a dual-layer structure, with each layer targeting a specific stage of wound healing (Figure 3A). Composed of gelatin and MPDA-lysozyme nanoparticles, the inner layer is devised to combat inflammation and infection triggered by biofilms. Given gelatin’s thermo-reversible transition from gel to sol, an increase in temperature facilitates the controlled release of nanoparticles. This allows the MPDA-lysozyme nanoparticles to penetrate biofilms during photothermal therapy (PTT). The outer layer is primarily constructed from gelatin methacryloyl (GelMA), which, once photo-crosslinked, provides a robust support to the wound, encouraging cell adhesion and growth. This layer also incorporates Epidermal Growth Factor (EGF) into GelMA, a strategic move to stimulate tissue regeneration and wound re-epithelization. The mesoporous structure of MPDA nanoparticles exhibits a larger surface area, which promotes efficient drug loading and delivery (Figure 3B). This facilitates loading a higher quantity of antibacterial agents onto MPDA nanoparticles, thereby enhancing the photothermal effect and compensating for the inadequacies of low-temperature photothermal therapy (PTT). When MPDA nanoparticles are loaded with lysozyme, they can effectively eliminate biofilms. The photothermal effect of MPDA nanoparticles disrupts the biofilms’ structure, enabling the loaded lysozyme to deeply penetrate the biofilms and exterminate bacteria (Figure 3C). Figure 3D demonstrates the efficacy of lysozyme-enhanced photothermal therapy by evaluating bacterial survival rate and wound pathology after treatment. Extremely low bacterial survival and lack of abscess in wounds treated with lysozyme-loaded nanoparticles plus laser validated the excellent antibacterial and antibiofilm effects. Hence, through this design, the hydrogel dressing provides an innovative, phased approach to wound care.

The author holds a positive view of their research findings. This dressing demonstrated the ability to effectively eliminate biofilms and promote the repair of chronic wounds. The bilayer hydrogel dressing utilized the thermo-reversible gelesol transition capability of gelatin to achieve a thermosensitive release of nanoparticles, allowing for deep penetration into biofilms and performing lysozyme-enhanced photothermal therapy to destroy biofilms. This study presents a promising and effective therapeutic strategy for addressing the challenging problem of chronic wound healing through the innovative use of a bilayer hydrogel dressing combined with PTT, suggesting its potential for clinical application.

### 3.3 Other types of hydrogels

Presently, various natural and synthetic hydrophilic polymers have been utilized to fabricate hydrogels with optimized parameters like porosity, swelling ratio and adhesion strength to benefit wound recovery (Wang et al., 2023b). Lysozyme is often incorporated into the 3D hydrogel matrix through blending or covalent conjugation (Wu et al., 2018; Lai et al., 2023). The hydrated environment and anti-adhesive properties enable hydrogels to facilitate re-epithelization while lysozyme provides antibacterial effects (Chen et al., 2022b). *In vitro* antimicrobial testing in simulated infected wound fluids and *in vivo* studies in animal wound models are performed to systematically evaluate the dual therapeutic functions. Composite designs with antimicrobial nanoparticles are explored for synergistic infection inhibition. The hydrogels demonstrate good biocompatibility, pronounced infection control and accelerated tissue repair (Ma et al., 2011). Their clinical translation potential as novel antibacterial and pro-healing wound dressings has been discussed (Omali et al., 2015; Banerjee, 2021). In summary, hydrogels offer a promising carrier system to deliver lysozyme for combating wound infections and regenerating healthy tissues.

Still, several challenges persist that need to be addressed in the application of hydrogels for the delivery of lysozyme in wound healing: 1) Control and optimization of lysozyme loading and release dynamics: The precise regulation and optimization of lysozyme loading capacity, along with its release kinetics, is a crucial aspect that demands further investigation. The heterogeneity among wound types and varying levels of infection necessitates a distinct and customized release dynamic for each case (Subbaraman et al., 2006). 2) Harmonizing hydrogel degradation rate with wound healing progression: A significant challenge lies in aligning the degradation rate of hydrogels with the rate of wound healing. A rapid degradation of the hydrogel could potentially hamper the wound healing process (Lee et al., 2009; Chaudhary et al., 2020; Chen et al., 2022b). Hence, it is imperative to ensure that the degradation pace of hydrogels does not supersede the tissue regeneration process.

## 4 Nanofilms

Nanofilms are ultra-thin layers of material, often only a few nanometers thick, which are engineered at the molecular level (Feng et al., 2017; Xu et al., 2018). Due to their thinness, they possess unique properties such as high transparency and a large surface area-to-volume ratio (Ding et al., 2020). These characteristics make them attractive for a wide array of applications, from electronics and optics to medicine and biotechnology. Nanofilms can be engineered to have precise thickness and uniformity, which allows for an accurate control over the dosage and release rate of lysozyme (Tian et al., 2021). Their large surface area enables high loading capacity of lysozyme. Additionally, nanofilms can adhere closely to the wound surface, ensuring direct delivery of the enzyme to the wound site (Xu et al., 2018; Chen et al., 2021). Furthermore, due to their thin and flexible nature, nanofilms may cause less discomfort to the patient compared to bulkier dressings. In terms of delivering lysozyme, both nanofilms and hydrogels have their unique advantages (Fang et al., 2021).

Nanofilms offer unique advantages for the delivery of lysozyme in wound healing due to their engineered structure (Fang et al., 2022). Their molecular-level precision allows for controlled and sustained release of the enzyme, providing a steady therapeutic effect. The large surface area-to-volume ratio of nanofilms supports a high loading capacity, meaning a small nanofilm can deliver a significant amount of lysozyme (Ding et al., 2020; Fu et al., 2022). The ability of nanofilms to adhere directly to the wound surface ensures maximal efficacy of the lysozyme, while their flexibility enhances patient comfort and effective coverage of the wound area. Additionally, the dense molecular packing within nanofilms can act as a barrier against bacteria, lowering the risk of infection, and their transparency facilitates continuous monitoring of the wound healing process (Saylan et al., 2017; Fu et al., 2022).

**FIGURE 4:**
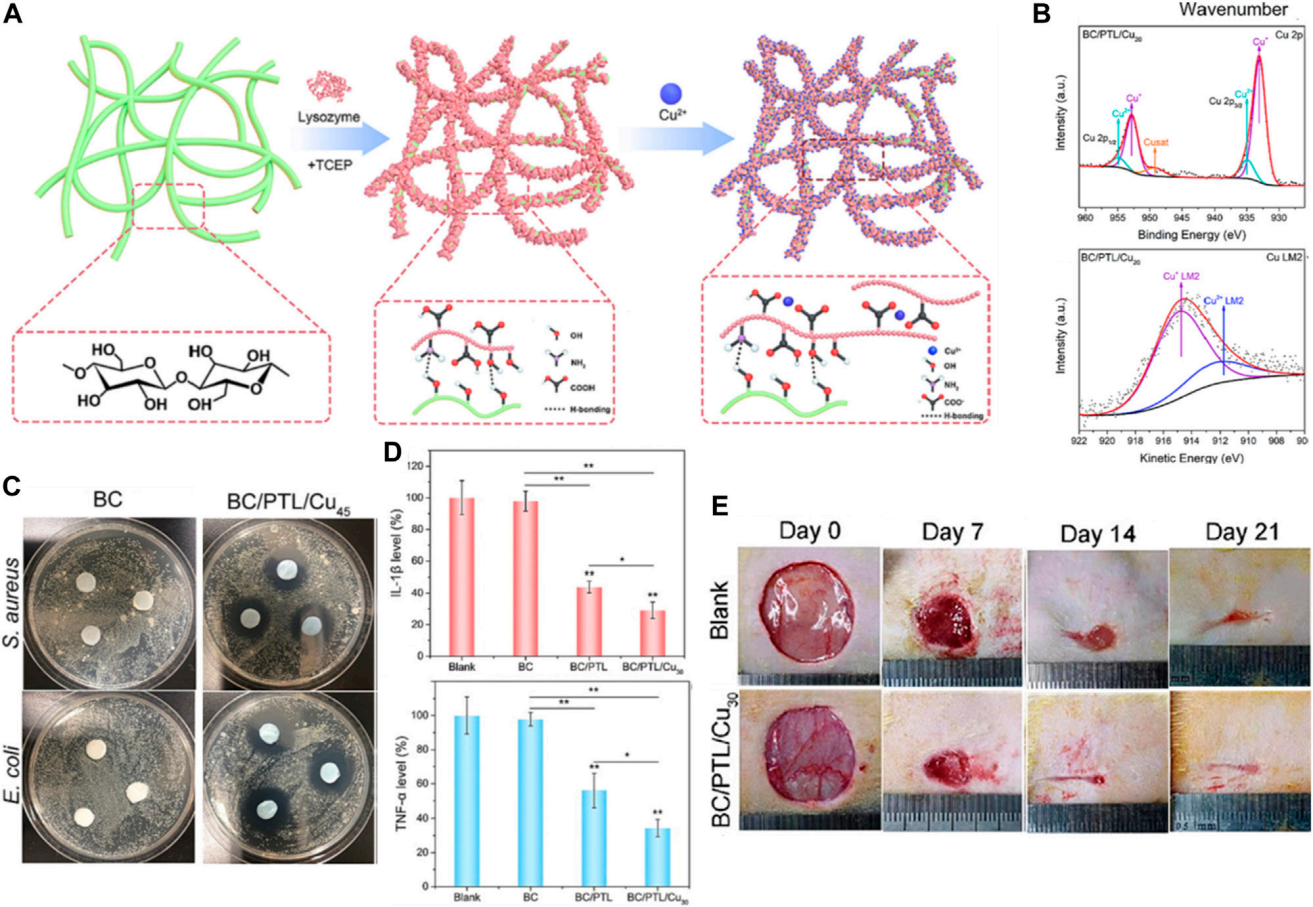
**(A)** Schematic illustration of the fabrication process for the BC/PTL/Cu composite materials. **(B)** Fourier Transform Infrared (FTIR) spectra and X-ray Photoelectron Spectroscopy (XPS) survey spectra for BC/PTL/Cu. **(C)** Photographs depicting the antibacterial activity of various samples, as evidenced by the inhibition zones against *S. aureus* and *E. coli*. **(D)** Quantitative analysis of Interleukin-1 beta (IL-1β) levels and tumor necrosis factor-alpha (TNF-α) levels. *p < 0.05, **p < 0.01. **(E)** Sequential photographs of wound healing progression on days 0, 7, 14, and 21 post-operations for the blank and BC/PTL/Cu30 groups. Reproduced with permission from Fu et al. (2022).

He et al. (2023) report the development of copper ion-incorporated lysozyme nanofilms fabricated on bacterial cellulose as antibacterial, anti-inflammatory and pro-angiogenic dressings for infected wound healing applications. The authors first prepared self-assembled lysozyme nanofilms on bacterial cellulose membranes via a facile heat-induced phase transition process. Copper ions were then loaded into the nanofilms through electrostatic interactions to impart antibacterial functionality (Figure 4A). Physicochemical characterization verified the successful construction of copper-lysozyme nanofilms on the cellulose matrix without compromising the mechanical integrity and cytocompatibility of the cellulose membranes (Figure 4B). The composite nanofilms exhibited strong antibacterial effects against both Gram-positive bacteria (*Staphylococcus aureus*) and Gram-negative bacteria (*E. coli*) attributed to the incorporated copper ions (Figure 4C). This addressed the risk of bacterial infection which is detrimental to wound healing. Anti-inflammatory effects were validated by the downregulation of TNF-α and IL-1β levels in rat wound tissues *in vivo* (Figure 4D). This helped resolve excessive inflammation that delays healing. In the infected rat wound model, the copper-lysozyme nanofilms significantly accelerated wound re-epithelialization and closure compared to bacterial cellulose alone, indicating its therapeutic efficacy in healing contaminated wounds (Figure 4E). The innovation of this work lies in the composite nanofilm-modified cellulose matrix approach to integrate multiple therapeutic actions to synergistically tackle various impediments of infected wound healing, including eliminating bacteria, resolving inflammation and promoting vascularization in one dressing material. This is superior to simply embedding single agents like antimicrobials into wound dressings.

Various nanofabrication methodologies, including layer-by-layer assembly, electrospinning, and templating, have been employed to create lysozyme-infused nanofilms (Liu et al., 2018; Zhou et al., 2023). A spectrum of materials, encompassing natural polymers such as chitosan, silk fibroin, and antimicrobial peptides, have been scrutinized for their applicability in crafting nanofilms with the requisite attributes (Liu et al., 2013). These nanofilms are frequently amalgamated with substrates like nanocellulose, meshes, and hydrogels to manufacture suitable wound dressing materials. Coupled with loading lysozyme, collaborative antibacterial effects are examined by integrating silver nanoparticles and other antimicrobials (Wang et al., 2017). The evaluation of anti-infective and pro-healing capacities is conducted by simulating infected wound conditions both *in vitro* and in animal models. A rational delivery system design emphasizes controlled release kinetics and the preservation of lysozyme activity. The potential for clinical translation of these nanocomposite dressings is a subject of ongoing discussion (Tenovuo, 2002). In conclusion, these studies demonstrate the versatility of nanofilm materials and synergistic antibacterial strategies, offering valuable insights for the optimized design of lysozyme nanofilm-based wound dressings.

Despite the promising prospects, the delivery of lysozyme using nanofilms for wound healing treatments presents several challenges. First, the fabrication of nanofilms with the desired properties and lysozyme loading necessitates the optimization of materials and nanofabrication techniques (Wu et al., 2020). Second, the preservation of lysozyme’s bioactivity during nanofilm creation and storage presents a significant hurdle, given the risk of enzyme denaturation or inactivation (Ohno and David, 1989; Omali et al., 2015). Achieving a sustained release of bioactive lysozyme in synergy with other antimicrobials is crucial, yet formidable. Furthermore, the nanofilms must possess mechanical durability for application while simultaneously permitting the permeation of nutrients and cell migration (DesJardins-Park et al., 2022). Biocompatibility and biosafety are additional factors to consider for clinical application. Lastly, controlled *in vivo* studies in infected animal wound models are indispensable to provide compelling evidence of antibacterial efficacy and wound healing promotion before initiating human trials. Additional efforts to understand nanofilm degradation, optimize therapeutic effects, and facilitate regulatory approval and commercialization are warranted (Frykberg Robert and Banks, 2015; Tian et al., 2022). In summary, the actualization of the full potential of lysozyme nanofilms in managing infected wounds remains a scientific challenge and is yet to be proven clinically. Collaborative efforts between material scientists, clinicians, and industry partners are paramount to surmount these obstacles.

## 5 Electrospun fibrous membranes

Electrospun fibrous mats are emerging as promising carriers for delivering lysozyme to treat infected wounds and facilitate healing (Charernsriwilaiwat et al., 2012; Huang et al., 2012). Typically fabricated by electrospinning natural or synthetic polymer solutions into nanoscale fibers and collecting them as porous mats, these fibrous membranes provide several favorable properties as lysozyme delivery platforms.

Firstly, the ultrafine fibers formed through electrostatic stretching provide an extremely high surface area to volume ratio, allowing high loading capacity for lysozyme (Huang et al., 2012). Secondly, the interconnected pore structure enables efficient nutrient transport, waste removal, and cell infiltration critical for tissue regeneration (Li et al., 2008). Thirdly, the nanotopography and biomimetic extracellular matrix of electrospun mats are beneficial for cell adhesion and growth. Fourthly, sustained release of lysozyme can be achieved by engineering the fiber matrix compositions and structures (Carvalho et al., 2022). Finally, electrospun mats can be directly used as wound dressings due to their flexibility, gas permeability and biocompatibility (Tonglairoum et al., 2015). *In vitro* and *in vivo* studies have shown electrospun lysozyme delivery systems exhibit pronounced antibacterial effects, improved re-epithelialization and collagen deposition compared to traditional wound dressings. With further optimization in large-scale manufacturing, electrospun antibacterial mats delivering lysozyme could be a competitive technology for advanced wound care.

**FIGURE 5:**
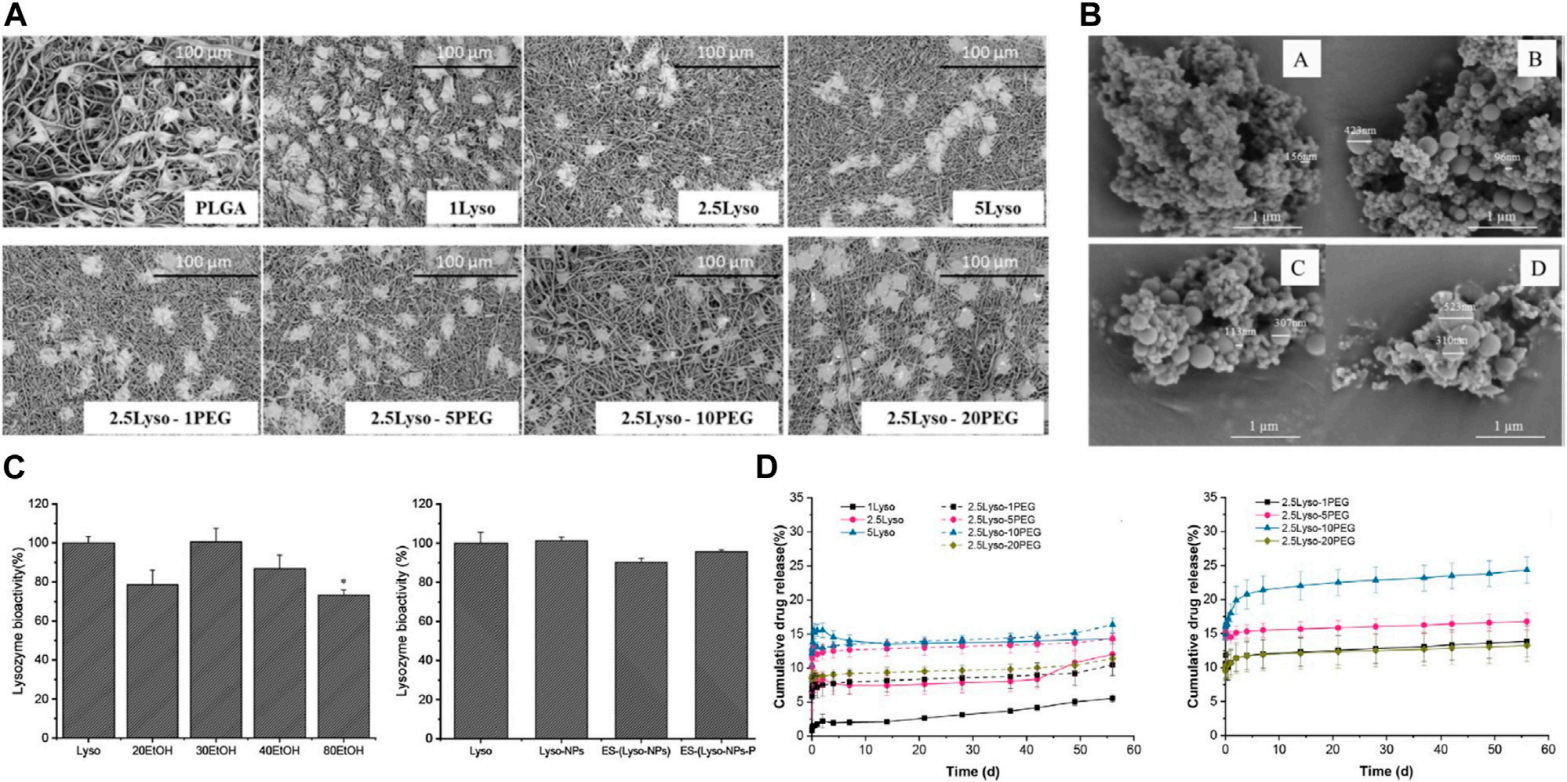
**(A)** SEM images showcase L929 cells propagated on Electrospun Fiber Mats (EFMs), both with and without the incorporation of lysozyme and PEG. **(B)** SEM images depicting electrosprayed Lyso-NPs derived from binary solvents with varying ratios of ethanol (EtOH) and Water (H_2_O). (A) 20:80 (v/v) EtOH-H_2_O, (B) 30:70 (v/v) EtOH-H_2_O, (C) 40:60 (v/v) EtOH-H_2_O, and (D) 80:20 (v/v) EtOH-H_2_O **(C)** Bioactivity of lysozyme, either electrosprayed or electrospun. **(D)** Release profile of lysozyme from EFMs in Phosphate-Buffered Saline (PBS) at pH 7.4 at 37°C and room temperature. Data represents mean ± SD, *n* = 3. Reproduced with permission from Carvalho et al. (2022).

Liu et al. (2019) report an innovative approach to incorporate lysozyme into electrospun fibrous matrices for sustained delivery and wound healing applications. The authors first engineered the nanofibrous mats by electrospinning a blended solution of silk fibroin, chitosan and lysozyme. By optimizing the electrospinning parameters and polymer ratios, uniform fibers in the nano-to-micro scale were produced with lysozyme dispersed in the protein and polysaccharide matrix (Figures 5A, B). A key objective was evaluating and ensuring the stability of lysozyme during the electrospinning process and subsequent storage, as the enzyme can be easily denatured or inactivated. Through comprehensive physicochemical characterization, the authors demonstrated that the presence of chitosan could significantly improve lysozyme stability compared to silk fibroin alone, owing to the favorable biocompatibility and anti-denaturation effects of the polysaccharide. An investigation into the bioactivity of lysozyme was conducted on electrosprayed Lyso-NPs. The results revealed a decline in bioactivity as the EtOH concentration in the binary solvent increased, relative to the raw lysozyme material (Figure 5C). The lysozyme-loaded mats were then subjected to release studies by immersing in a simulated wound fluid environment. Sustained release of bioactive lysozyme was achieved for over 4 weeks (Figure 5D). Furthermore, the mats showed enhanced cell adhesion and proliferation capacities *in vitro* compared to traditional wound dressings like gauze. This illustrated their cytocompatibility and pro-healing properties.

The key significance and novelty of this work lies in the successful development of an electrospun enzyme delivery system using a synergistic silk fibroin/chitosan matrix to stabilize lysozyme activity. This provides valuable insights to guide further optimization of electrospinning parameters and material compositions for fabricating multifunctional lysozyme-eluting wound dressings.

Electrospun fibrous membranes have shown promise as carriers for lysozyme delivery to treat wound infections and facilitate healing (Yang et al., 2008; Liu et al., 2019). However, there are still challenges that need to be addressed before their widespread clinical translation: firstly, scale-up manufacturing of uniform fibrous matrices with reproducible properties remains a challenge (Raheja et al., 2013). The electrospinning process requires meticulous optimization and control of multiple parameters such as solution viscosity, flow rate and electric field strength to generate fibers at the nano- to micro-scale. Maintaining consistency in fiber morphology and dimensions during large-scale production is difficult (Bugatti et al., 2018). Secondly, effective encapsulation and stabilization of lysozyme activity within the electrospun fibers needs further improvement. Lysozyme is prone to denaturation and loss of enzymatic function during the electrospinning process due to shear stress and interactions with solvents. Strategies to prevent inactivation warrant further studies. Thirdly, achieving sustained release of bioactive lysozyme over clinically relevant timescales has not been adequately demonstrated (Chaudhary et al., 2020; Carvalho et al., 2022). The release kinetics can be tailored by the fiber matrix composition and treatment but optimal designs are still empirical. Fourthly, *in vivo* efficacy and safety evaluations are still limited. More preclinical studies in infected animal wound models are imperative to provide solid evidence to support their clinical use.

Future research is needed to better understand the interplay between electrospun fiber properties and lysozyme release kinetics, stability and functionality (Chiu et al., 2011; Puhl et al., 2014). Novel biofunctionalization techniques, combination delivery with other antimicrobials, and mechanistic studies on synergistic biofilm disruption and tissue regeneration will enable further optimization delivery systems (Briggs et al., 2015; Wu et al., 2021; Zhao et al., 2022). With advances in scalable nanomanufacturing, electrospun mats carrying lysozyme could become a competitive technology for advanced antibacterial wound care.

## 6 Modified-lysozyme composite

Lysozyme-biomacromolecule composites are structures where the antimicrobial enzyme lysozyme is integrated into or bonded with a biomacromolecule (De France et al., 2020). These biomacromolecules could be naturally occurring substances like proteins or polysaccharides, or even synthetic biological substances such as certain polymers (de Oliveira et al., 2012). The use of lysozyme-biomacromolecule composites for lysozyme delivery carries several advantages. For instance, the biomacromolecule can provide a protective shell to the lysozyme, shielding it from degradation in harsh environments and thus preserving its bioactivity (Park et al., 2004). The biomacromolecules also offer a way to control the release of lysozyme over time. This release can be fine-tuned according to the properties of the biomacromolecule and the specific therapeutic needs (Li et al., 2017).

Moreover, many biomacromolecules exhibit biocompatibility and biodegradability. This means they can be safely introduced into the body where they will eventually break down into non-harmful byproducts (Agarwal et al., 2017). Further, the biomacromolecule can be functionalized to target specific cells or tissues, thus enhancing the effectiveness of the lysozyme. Consequently, the use of these composites can enhance lysozyme delivery and effectiveness, potentially reducing the required dosage and associated side effects (Branchu et al., 1999). Therefore, these composites represent a promising platform for efficient and safe lysozyme delivery in various therapeutic applications.

**FIGURE 6:**
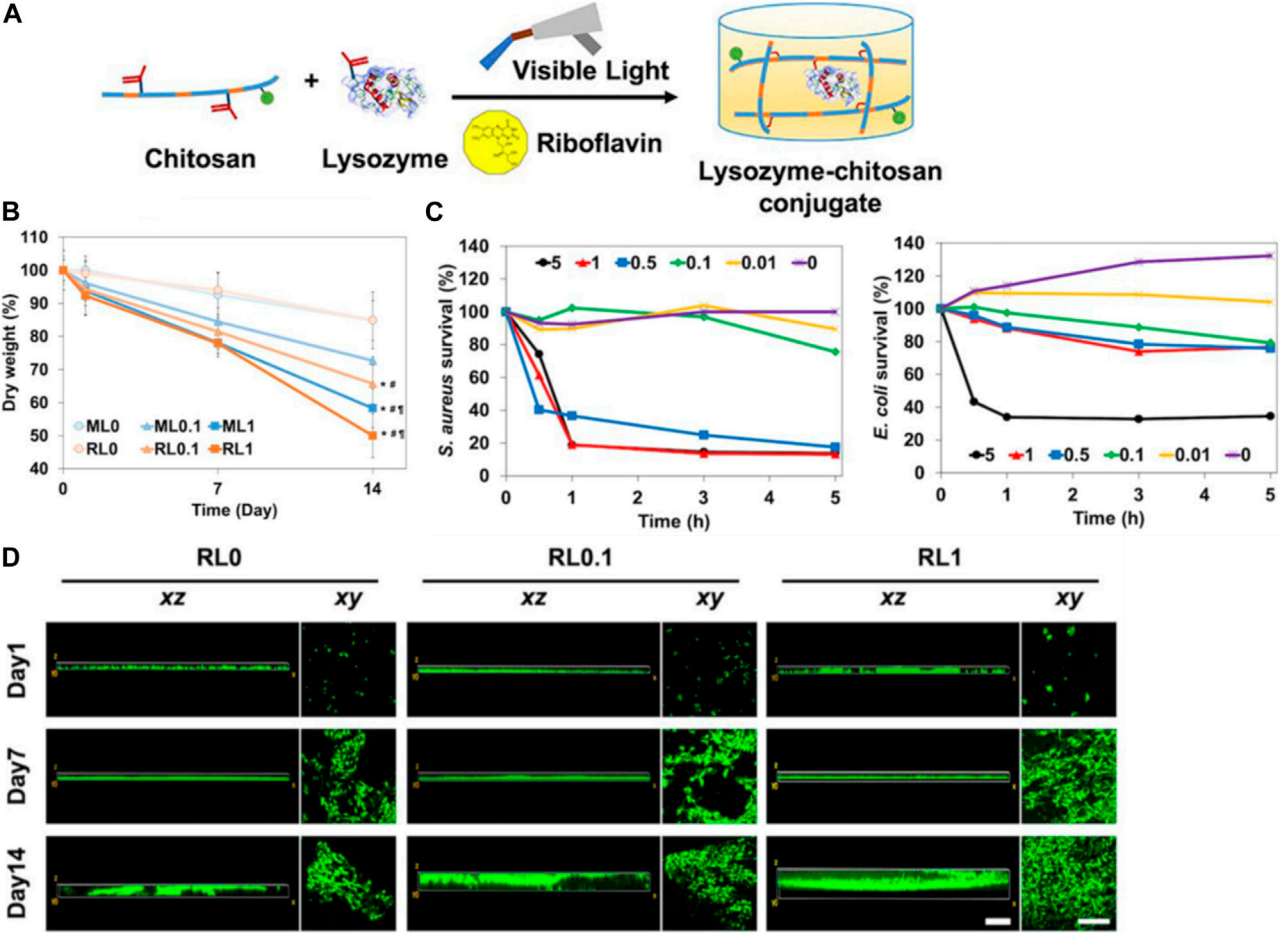
**(A)** Diagram illustrating the dual-functional hydrogel composed of lysozyme-chitosan conjugate. **(B)** The 2-week degradation pattern as determined by changes in the dry weight of the hydrogels. **(C)** Assessment of *in vitro* antibacterial activity of free lysozyme at varying concentrations over a period of 5 h. **(D)** *In vitro* observation of NIH/3T3s cell migration on hydrogels over a 2-week period, assessing movement in both vertical (xz) and horizontal (xy) directions. Reproduced with permission from Park et al. (2004).

For example, Kim et al. (2020) construct an innovative lysozyme-chitosan conjugate system with dual antibacterial and tunable degradability properties for infected wound healing. Lysozyme was covalently linked to chitosan chains using a hydrolysable linker (Figure 6A). In comparison to free lysozyme, the obtained conjugates exhibited enhanced thermal and storage stability. The conjugates could release active lysozyme in a tunable manner dependent on the linker, with over 80% activity retained after release (Figure 6B). The released lysozyme showed significant bactericidal effects against both gram-positive and gram-negative bacteria (Figure 6C). The incorporation of lysozyme into the chitosan-hydrogels appears to enhance cell migration and spreading and lysozyme could potentially enhance cell infiltration and tissue integration when used in lysozyme-chitosan conjugate system (Figure 6D). This lysozyme-conjugate system integrates antibacterial lysozyme delivery in a cytocompatible and tunably degradable chitosan platform. It represents a promising bioactive dressing for infected wound management.

The application of lysozyme-biomacromolecule complexes as delivery vehicles for lysozyme in wound healing therapy holds promising prospects, but it also faces several significant challenges. One major issue is to attain a well-regulated and sustained release of lysozyme. A swift release might lead to an initial burst of lysozyme at the wound site, which subsequently diminishes over time, potentially reducing the therapeutic effectiveness (Arcan and Ahmet, 2013). Furthermore, maintaining the stability and activity of lysozyme within the complex is crucial. As a protein, lysozyme is prone to denaturation or degradation, compromising its antibacterial properties. Designing a carrier that can safeguard lysozyme from such degradation, especially considering the harsh conditions of a wound environment, is a key challenge. In addition, the biocompatibility and safety of the lysozyme-biomacromolecule complex are essential. Although biomacromolecules are generally biocompatible, the specific characteristics of the complex, the potential for immune reactions, and the consequences of degradation products on surrounding tissues need thorough evaluation (Stawski et al., 2019).

From a production standpoint, the process development for manufacturing these complexes is another hurdle (Morton and Tania, 2016; Wu et al., 2022). The production should be reproducible, scalable, and cost-effective, making it practical for widespread clinical use (Brunaugh et al., 2021). With the advent of personalized medicine, the development of customized lysozyme-biomacromolecule complexes that cater to individual patient’s needs could be an interesting future direction (Nganga et al., 2012). Furthermore, extensive clinical trials are needed to evaluate the safety and efficacy of these complexes in human patients and to optimize treatment protocols (Lee et al., 2010). Despite these challenges, the potential benefits of lysozyme-biomacromolecule complexes in wound healing warrant continued research and development.

## 7 Conclusion and perspectives

Given the challenges of maximizing the therapeutic potential of lysozyme, several innovative delivery systems have been developed, including hydrogels, nanofilms, electrospun fibrous membranes, and modified-lysozyme composite systems (Yang et al., 2008; Wang et al., 2023a; He et al., 2023). These platforms not only enhance the stability of lysozyme and reduce its immunogenicity, but also extend its retention at wound sites by enabling a sustained release, thereby prolonging its antibacterial action and mitigating rapid degradation (Wang et al., 2017). Harnessing the therapeutic potential of lysozyme through various delivery systems indeed offers promising prospects (Xu et al., 2018; Xie et al., 2022). However, these systems also present several challenges that need to be addressed to fully exploit their potential.

The stability of lysozyme within these delivery systems is another significant challenge. Being a protein, lysozyme is susceptible to denaturation or degradation, which could decrease its antibacterial properties. The delivery system must hence protect lysozyme from degradation while maintaining its activity (De Vita et al., 1984). The biocompatibility and safety of these delivery systems are also of utmost importance (Nunan et al., 2014). Although the materials used are generally considered biocompatible, the specific composite nature of the systems, the potential for immune reactions, and the effect of degradation products on surrounding tissues must be thoroughly evaluated (Ragland and Alison, 2017; Zou et al., 2019). From a production point of view, the fabrication processes of these delivery systems pose another challenge. The production should be scalable, reproducible, and cost-effective, making it practical for widespread clinical application (Swaminathan et al., 2011; Woods et al., 2011). Last but not least, the clinical validation of these delivery systems is a significant challenge. More extensive *in vivo* studies and clinical trials are needed to confirm their safety, efficacy, and performance in a real-world setting.

However, each of these delivery systems has its own limitations and challenges, such as the complexity of fabrication, potential loss of lysozyme activity during processing, and the need for further clinical validation (Gudipaneni et al., 2014; Omali et al., 2015). Despite these challenges, the advancements in these delivery systems have opened up new avenues for the therapeutic application of lysozyme in wound healing (Gong et al., 2022; Khalil and Mohammad, 2023).

In conclusion, the use of lysozyme-loaded delivery systems, including hydrogels, nanofilms, electrospun fibrous membranes, and modified-lysozyme composite systems, represents an exciting advancement in wound healing applications. The future of lysozyme in wound healing indeed looks promising, with a range of different delivery systems being developed to exploit its therapeutic potential. However, several challenges, particularly concerning the controlled release, stability of lysozyme, biocompatibility, safety, and clinical validation of these systems, need to be addressed. Continued research and innovation, particularly focusing on the effective delivery of beneficial ions, are vital for overcoming these challenges. By doing so, the field can move closer to fully realizing the therapeutic potential of lysozyme, ultimately leading to improved wound care strategies and better patient outcomes.
